# Comparing adolescent glomerular disease clinical outcomes to the clinical outcomes in childhood, young adult, and adult-onset glomerular disease in the CureGN database

**DOI:** 10.1007/s00467-024-06566-4

**Published:** 2024-12-27

**Authors:** Kelly Garrity, Nathaniel Putnam, Elaine S. Kamil, Susan Massengill, Myda Khalid, Rachana Srivastava, Jaya Isaacs, Eloise Salmon

**Affiliations:** 1 Mattel Department of Pediatrics, David Geffen School of Medicine at UCLA, Los Angeles, CA USA; 2 Medical Univerity of South Carolina, Charleston, SC USA; 3 University of Michigan School of Medicine, Ann Arbor, MI USA; 4 Cedars-Sinai Medical Center, David Geffen School of Medicine at UCLA, Los Angeles, CA USA; 5 Levine Children’s Hospital, Charlotte, NC USA; 6 Riley Hospital for Children, Indiana University School of Medicine, Indianapolis, IN USA; 7 Montefiore Children’s Hospital, Albert Einstein School of Medicine, Bronx, NY USA

**Keywords:** Adolescent, Glomerular disease, CureGN, Minimal change disease, Focal glomerulosclerosis, IgA nephroapthy, Membranous nephropathy, CKD

## Abstract

**Background:**

There is a lack of evidence to suggest that outcomes of adolescent and adult-onset glomerular disease differ. Still, most glomerular disease trials include adults but exclude adolescents.

**Methods:**

We designed a retrospective study using the CureGN database to compare individuals with adolescent-onset glomerular disease relative to individuals with older and younger age at onset. The two main outcomes were sustained proteinuria remission off immunosuppression treatment and composite eGFR decline.

**Results:**

Our data did not show a significant difference in sustained proteinuria remission off treatment or composite eGFR decline between adolescent onset glomerular disease and either childhood (age 5–12), young adult (age 20–29), or adult (age 30–39) onset glomerular disease. Having high-risk *APOL1* alleles and hypertension at the time of study enrollment decreased the likelihood of achieving sustained proteinuria remission off treatment. While participants with minimal change disease and IgA nephropathy were similarly likely to achieve sustained proteinuria remission off treatment, participants with focal segmental glomerulosclerosis and membranous nephropathy were less likely to achieve sustained proteinuria remission off treatment compared to participants with minimal change disease. CKD stage, high-risk *APOL1* alleles, hypertension stage, and education all significantly impacted the likelihood of progression to the composite eGFR decline outcome.

**Conclusions:**

Approximately 25% of each age cohort reached the composite eGFR decline outcome within 5 years. As more glomerular disease clinical trials become available, we must consider opening these trials to people with childhood and adolescent onset disease since like adults they are at high risk of progressive kidney function decline.

**Graphical abstract:**

**Figure:**
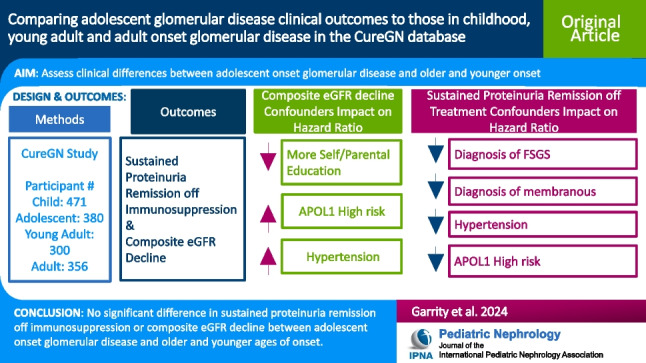
A higher resolution version of the Graphical abstract is available as Supplementary information

**Supplementary Information:**

The online version contains supplementary material available at 10.1007/s00467-024-06566-4.

## Background

While many studies aggregate adolescent onset glomerular disease with childhood onset disease, there are data to suggest adolescent onset glomerular disease outcomes may mirror adult-onset outcomes more closely than younger childhood onset outcomes. The only study to our knowledge addressing adolescent onset glomerular disease outcomes found that survival without kidney failure at 1 and 5 years for glomerulopathies, and both minimal change disease (MCD) and focal segmental glomerulosclerosis (FSGS) specifically, were not statistically different for adults vs. adolescents [1]. Meanwhile, there is some evidence to suggest differences between adolescent and pediatric onset glomerular disease. Although approximately 75% of childhood nephrotic syndrome (NS) presents as a MCD picture, much like in adulthood, a FSGS presentation is more common in adolescent [1–3]. Prior pediatric data have suggested individuals with IgA nephropathy (IgAN), FSGS, and membranous nephropathy (MN) have better clinical outcomes if disease onset occurred earlier in childhood [4–9]. Some have suggested that the change in hormones associated with puberty may result in a decline in kidney function [10]. Another explanation may be that younger individuals have healthier kidney tissue at the time of disease onset. Among carefully screened healthy kidney donors, glomerular filtration rate (GFR) declines at a rate of 6.3 mL/min/1.73 m^2^ per decade [11]. Additionally, others have suggested that younger age may be associated with more estimated GFR (eGFR) recovery due to a different response to immune stimuli across different age groups [12].

Whether adolescent onset glomerular disease is like adult onset glomerular disease has become increasingly relevant as the number of interventional glomerular diseases trials have increased significantly in the last 10 years. Unfortunately, the majority of the studies are restricted to adults and there is evidence that the percentage of studies restricted to adults is only increasing [13]. Despite the median age of an IgA diagnosis being 40, one analysis found that 83% of IgAN clinical trials excluded patients under age 18, while only 7% excluded adults over age 65 years old [14, 15].

Given a lack of clarity as to whether adolescent onset glomerular disease outcomes are like younger children or adults and the possibility adolescents may have an entirely different disease trajectory/outcomes, our objective was to assess the clinical outcomes of adolescent onset glomerular disease and compare that to older and younger age groups in the CureGN cohort.

## Methods

### Study population

Our study population was derived from the CureGN study database. CureGN is a multi-center, prospective observational cohort study of patients with glomerular disease funded by the NIH-NIDDK. CureGN enrolls children and adults within 5 years of a first clinically indicated kidney biopsy, demonstrating MCD, FSGS, MN, or IgA nephropathy/vasculitis (IgAN/IgAV) from the USA, Canada, Italy, and Poland. Individuals with kidney failure, diabetes, or malignancy prior to enrollment are excluded. Design details have been published previously [16]. Patients in CureGN are treated at their local nephrologist’s discretion based on their unique clinical characteristics. Our study population consisted of 1507 Cure GN participants between ages 5 and 39 years of age at the time of biopsy or diagnosis, prioritizing the earliest date. Twelve percent of patients enrolled outside the United States.

### Derived variables

Our exposure of interest was age at diagnosis, categorized as child onset age 5–12 years, adolescent onset age 13–19 years, young adult onset age 20–29 years, and adult onset age 30–39 years of age. We had two main outcomes, with the first being sustained proteinuria remission off immunosuppression treatment, defined as a UPCR < 0.3 mg/mg and/or UA protein that was negative or trace for at least 1 year while off immunosuppressive therapies with ≥ 75% preservation of the eGFR at the time of biopsy, and participants having maintained an eGFR greater than 25 mLs/min/1.73 m^2^ and censored at the time of kidney failure [17, 18]. We created the sustained proteinuria remission off treatment measure to capture prolonged clinical disease remission, which did not place the patient at risk for the adverse effects of immunosuppression. We used the CKD-EPI 2021 equation to calculate eGFRs for patients > 25 years old and used the CKIDU25 equation to calculate eGFRs for patients 25 years or younger [19, 20]. All eGFRs over 120 mLs/min/1.73 m^2^ were windsorized to 120 mLs/min/1.73 m^2^. The IgAN cohort also required a urine dipstick blood negative or trace during follow-up [21, 22].

The second outcome was composite eGFR decline. We defined composite eGFR decline as requiring chronic kidney replacement therapy or an eGFR < 15 mLs/min/1.73 m^2^ and/or extrapolated 40% reduction in eGFR since biopsy resulting in an observed eGFR < 60 mLs/min/1.73 m^2^ [23].

We utilized the American Academy of Pediatrics 2017 Blood Pressure guidelines to define hypertension stages in participants with childhood and adolescent onset glomerular disease and we utilized the American Heart Association 2017 Blood Pressure guidelines to define hypertension stages in participants with young adult and adult onset glomerular disease [24, 25]. We defined the stage of hypertension based on the participant’s blood pressure at the time of enrollment. Education level was fixed throughout the study and based on parental education at CureGN enrollment, prioritizing maternal education if available, in child and adolescent onset groups, and personal education level at CureGN enrollment diagnosis in young adult and adult-onset groups. Many coexisting conditions were aggregated into a summary variable as “the presence of any coexisting conditions,” and a list of the relevant CureGN survey questions can be found in Supplementary Table S1. All participants enrolling in CureGN were offered genetic testing with whole-genome sequencing, with not all participants opting in. The presence of G1/G1, G2/G2, or G1/G2 alleles in the *APOL1* gene was classified as high risk [26]. Participants who had not consented to genetic testing were placed in the “unknown risk” category.

Medication adherence was tracked using CureGN survey questions. During the first five years of the CureGN study, medication adherence was assessed using three questions, answered on a Likert scale from 1 to 5, with 1 meaning strongly disagree and 5 meaning strongly agree: “I took all doses of my disease medication”; “I missed or skipped at least one dose of my kidney disease medication”; and “I was not able to take all of my kidney disease medication”. Medication nonadherence was defined as an answer of neutral to any disagreement (1, 2, or 3) to the first question or an answer to neutral or any agreement (3, 4, or 5) to the second or third questions. After 2020, revisions to the CureGN protocol medication adherence questions were implemented as follows: participants were asked only the following two questions: “Are you careless at times about taking your medicine” and “Do you ever forget your medicine.” Medication nonadherence was defined as an affirmative response to either of these questions.

### Statistical analysis

Descriptive statistics and demographic characteristics were calculated for the study population. To analyze the risk of the composite eGFR decline outcome in different onset groups, we used a Cox proportional hazards time-to-event model initially adjusted for sex, race, ethnicity, education level, chronic kidney disease (CKD) stage at biopsy, glomerular disease diagnosis, medication adherence, the presence of any coexisting conditions, and APOL1 risk. Backwards variable selection was used to select variables for the final model. To analyze the risk of sustained proteinuria remission off treatment in different onset groups, we performed a second survival analysis, again initially adjusting for sex, race, ethnicity, education level, CKD stage at biopsy, glomerular disease diagnosis, medication adherence, the presence of any coexisting conditions, and APOL1 risk. Backwards variable selection was used to select variables for the final model. Unadjusted survival curves of both outcomes, stratified by both diagnosis and onset age group, were also plotted. Missing biomarkers were not imputed for this analysis. Analysis included patients enrolled in CureGN prior to December of 2023.

### Ethics

The study protocol was reviewed and approved at all enrolling sites during the initial 5 years and by the single IRB SALUS #20038 with the University of Michigan as the data coordinating center since 2018. All study participants or legal guardians provided informed consent and children of appropriate age provided informed assent, prior to any study procedures.

## Results

Demographic and clinical characteristics are provided in Table 1. Our study contained 1507 participants: 34% were non-white race, IgAN was the most represented diagnosis, median eGFR at biopsy was 93.7 mLs/min/1.73 m^2^, and median uPCR at biopsy was 2.4 mg/mg.

**Table 1:** Demographic and summary statistics of the study population

	Age at biopsy			
	5–12 (*n* = 471)	13–19 (*n* = 380)	20–29 (*n* = 300)	30–39 (*n* = 356)
Age at CureGN enrollment	10 (8, 12)	17 (15, 18)	26 (24, 29)	36 (34, 39)
Years of follow-up	4.9 (3.1, 6.1)	4.1 (1.7, 5.5)	4.1 (1.6, 6.0)	4.1 (1.6, 5.7)
Age at most recent visit	15.0 (12.7, 17.4)	20.8 (18.6, 22.9)	30.0 (27.5, 33.9)	40.4 (37.9, 43.5)
Time from biopsy to most recent visit	6.1 (4.7, 7.8)	5.1 (3.2, 6.7)	5.4 (3.0, 7.6)	5.3 (3.3, 7.5)
Sex				
Female	269 (57%)	226 (59%)	154 (51%)	198 (56%)
Male	202 (43%)	154 (41%)	146 (49%)	158 (44%)
Race:				
Black/African–American	69 (15%)	84 (22%)	45 (15%)	50 (14%)
White	323 (69%)	256 (67%)	182 (61%)	227 (64%)
Other	79 (17%)	40 (11%)	73 (24%)	79 (22%)
Ethnicity:				
Hispanic	63 (13%)	56 (15%)	52 (17%)	64 (18%)
Not Hispanic	406 (86%)	324 (85%)	245 (82%)	289 (81%)
Unknown	2 (0%)	0 (0%)	3 (1%)	3 (1%)
Diagnosis				
MCD	165 (35%)	70 (18%)	51 (17%)	41 (12%)
FSGS	108 (23%)	115 (30%)	84 (28%)	95 (27%)
MN	17 (4%)	40 (11%)	42 (14%)	79 (22%)
IgAN	173 (37%)	141 (37%)	109 (36%)	116 (33%)
APOL1 risk:				
High risk	13 (3%)	28 (7%)	15 (5%)	19 (5%)
Low risk	281 (60%)	211 (56%)	199 (66%)	222 (62%)
Unknown risk	177 (38%)	141 (37%)	86 (29%)	115 (32%)
Hypertension category at enrollment				
Normal blood pressure	268 (57%)	191 (50%)	104 (35%)	110 (31%)
Elevated blood pressure	57 (12%)	69 (18%)	34 (11%)	39 (11%)
Stage 1 hypertension	94 (20%)	71 (19%)	80 (27%)	84 (24%)
Stage 2 hypertension	22 (5%)	24 (6%)	52 (17%)	75 (21%)
Unknown	30 (6%)	25 (7%)	30 (10%)	48 (13%)
Ever diagnosed with hypertension:				
Ever	332 (70%)	240 (63%)	159 (53%)	156 (44%)
Never	131 (28%)	124 (33%)	125 (42%)	175 (49%)
Unknown	8 (2%)	16 (4%)	16 (5%)	25 (7%)
Median eGFR at biopsy (IQR)	109.3 (87.7, 131.2)	88.6 (73.2, 108.5)	87.3 (56.6, 114.9)	79.2 (48.9, 115.0)
Median UPCR at biopsy* (IQR)	2.2 (0.4, 6.3)	1.8 (0.5, 5.5)	2.9 (0.9, 6.0)	2.6 (0.9, 6.4)

Unless otherwise specified, continuous variables reported as median (25th,75th percentile)

*eGFR* estimated glomerular filtration rate in mLs/min/1.73 m^2^

^*^For median UPCR at Biopsy we used the UPCR collected closest to the time of biopsy

**Fig. 1:**
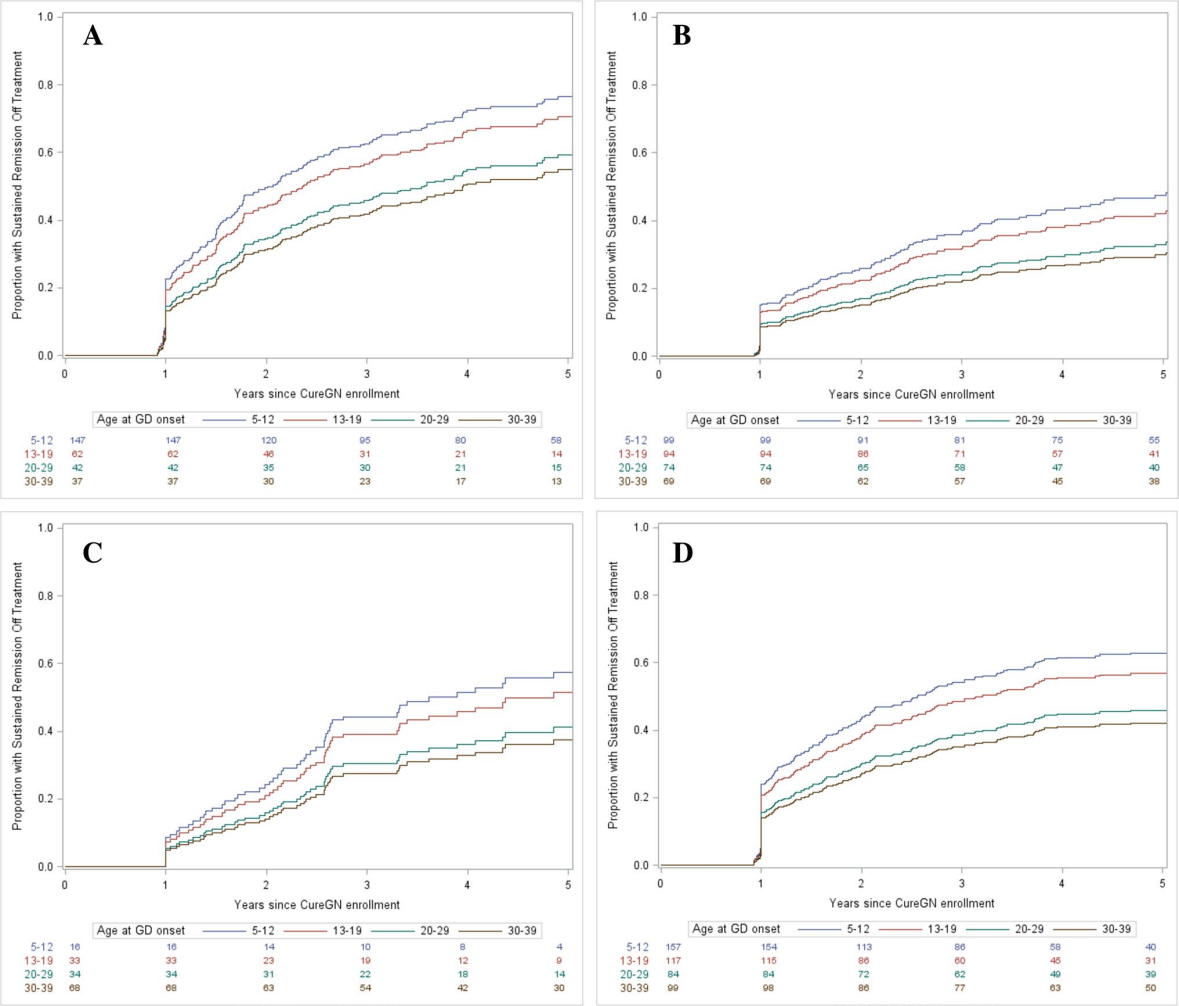
**A** Time to one year of sustained glomerular disease proteinuria remission off immunosuppressive therapy in patients with minimal change disease, by age (unadjusted). We defined sustained proteinuria remission off immunosuppression treatment as a UPCR < 0.3 and/or UA protein that was negative or trace for at least 1 year while off immunosuppressive therapies with ≥ 75% preservation of the eGFR at the time of biopsy, and participants having maintained an eGFR greater than 25 mLs/min/1.73 m^2^ and censored at the time of kidney failure. **B** Time to one year of sustained glomerular disease proteinuria remission off immunosuppressive therapy in patients with focal segmental glomerular sclerosis, by age (unadjusted). We defined sustained proteinuria remission off immunosuppression treatment as a UPCR < 0.3 and/or UA protein that was negative or trace for at least 1 year while off immunosuppressive therapies with ≥ 75% preservation of the eGFR at the time of biopsy, and participants having maintained an eGFR greater than 25 mLs/min/1.73 m^2^ and censored at the time of kidney failure. **C** Time to one year of sustained glomerular disease proteinuria remission off immunosuppressive therapy in patients with membranous nephropathy, by age (unadjusted). We defined sustained proteinuria remission off immunosuppression treatment as a UPCR < 0.3 and/or UA protein that was negative or trace for at least 1 year while off immunosuppressive therapies with ≥ 75% preservation of the eGFR at the time of biopsy, and participants having maintained an eGFR greater than 25 mLs/min/1.73 m^2^ and censored at the time of kidney failure. **D** Time to one year of sustained glomerular disease proteinuria remission off immunosuppressive therapy in patients with IgA nephropathy, by age (unadjusted). We defined sustained proteinuria remission off immunosuppression treatment as a UPCR < 0.3 and/or UA protein that was negative or trace for at least 1 year while off immunosuppressive therapies with ≥ 75% preservation of the eGFR at the time of biopsy, and participants having maintained an eGFR greater than 25 mLs/min/1.73 m^2^ and censored at the time of kidney failure with a urine dipstick blood negative or trace during follow-up

Participants with adolescent-onset glomerular disease had a similar likelihood of achieving sustained proteinuria remission off treatment as participants with child, young adult, and adult-onset glomerular disease. However, with each of the four glomerular diseases there was a trend in the unadjusted analysis for the younger the age of onset, the more likely the participants would achieve sustained proteinuria remission off treatment (see Fig. 1). As the IgAN measure for sustained proteinuria remission off treatment required hematuria and proteinuria remission, of the 516 IgAN participants, 218 achieved both the hematuria and the UPCR metric, while 120 patients met the hematuria metric alone, and 56 participants met the UPCR metric alone. Ethnicity, sex, race, education, presence of coexisting conditions, and medication adherence did not significantly impact the likelihood of achieving sustained proteinuria remission off treatment in this model. Participants with FSGS and MN were significantly less likely to achieve sustained proteinuria remission off treatment compared to participants with MCD. However, participants with IgAN did not have a significantly different likelihood of achieving sustained proteinuria remission off treatment than participants with MCD. Participants with a high-risk *APOL1* alleles were significantly less likely to achieve sustained proteinuria remission off treatment than participants with low-risk *APOL1 *alleles. Participants with stage 1 and stage 2 hypertension at the time of study admission were also significantly less likely to achieve sustained proteinuria remission off treatment than participants with a normal range blood pressure (see Table 2).

**Table 2:** Sustained proteinuria remission off treatment in children and young adults with glomerular disease multivariate model where a higher hazard ratio indicates more likely to experience remission

Sustained proteinuria remission off treatment model	Hazard ratio (CI)
Age at glomerular disease onset (reference: 13–19)	
5–12	1.14 (0.91, 1.43)
20–29	0.82 (0.63, 1.09)
30–39	0.78 (0.59, 1.03)
eGFR at biopsy (reference: eGFR 90 +)	
60–90	0.84 (0.67, 1.04)
30–60	0.58 (0.42, 0.79)
15–30	0.67 (0.40, 1.15)
< 15	1.06 (0.54, 2.07)
Diagnosis (reference: MCD)	
FSGS	0.56 (0.44, 0.72)
IgAN	0.81 (0.65, 1.00)
MN	0.56 (0.40, 0.78)
APOL1 risk (reference: low risk)	
High risk	0.53 (0.31, 0.91)
Unknown risk	1.14 (0.94, 1.40)
Hypertension status at enrollment (reference: normal blood pressure)	
Elevated blood pressure	1.07 (0.84, 1.37)
Stage 1 hypertension	0.72 (0.58, 0.90)
Stage 2 hypertension	0.50 (0.35, 0.717)

*MCD* minimal change disease, *FSGS* focal segmental glomerulosclerosis, *MN* membranous nephropathy, *IgAN* IgA nephropathy

**Fig. 2:**
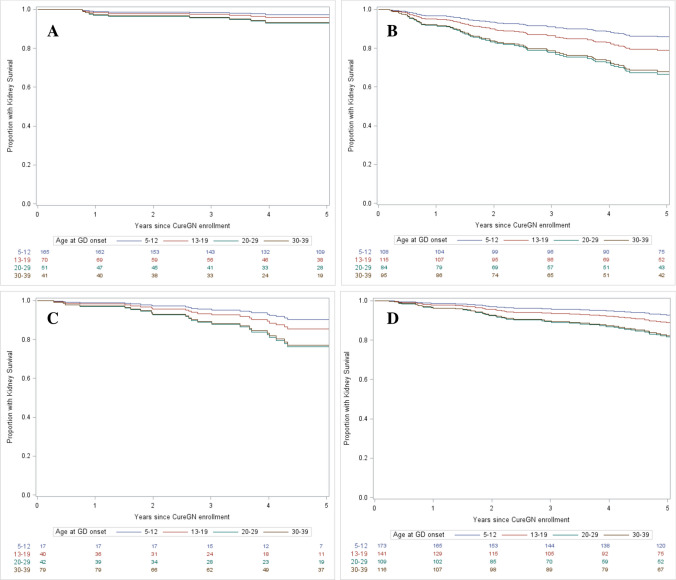
**A** Composite eGFR decline of children and young adults with minimal change disease, by age (unadjusted). We defined composite eGFR decline as requiring chronic kidney replacement therapy or an eGFR < 15 mLs/min/1.73 m^2^ and/or extrapolated 40% reduction in eGFR since biopsy resulting in an observed eGFR < 60 mLs/min/1.73 m^2^. **B** Composite eGFR decline of children and young adults with focal segmental glomerulosclerosis, by age (unadjusted). We defined composite eGFR decline as requiring chronic kidney replacement therapy or an eGFR < 15 mLs/min/1.73 m^2^ and/or extrapolated 40% reduction in eGFR since biopsy resulting in an observed eGFR < 60 mLs/min/1.73 m^2^. **C** Composite eGFR decline of children and young adults with membranous nephropathy, by age (unadjusted). We defined composite eGFR decline as requiring chronic kidney replacement therapy or an eGFR < 15 mLs/min/1.73 m^2^ and/or extrapolated 40% reduction in eGFR since biopsy resulting in an observed eGFR < 60 mLs/min/1.73 m^2^. **D** Composite eGFR decline of children and young adults with IgA nephropathy, by age (unadjusted). We defined composite eGFR decline as requiring chronic kidney replacement therapy or an eGFR < 15 mLs/min/1.73 m^2^ and/or extrapolated 40% reduction in eGFR since biopsy resulting in an observed eGFR < 60 mLs/min/1.73 m^2^

Participants with adolescent-onset glomerular disease did not have significantly different rates of progression to the composite eGFR decline outcome when compared to child-onset, young adult-onset, and adult-onset participants. However, with each of the four glomerular diseases, there was a trend in the unadjusted analysis for the younger the age of onset, the less likely the participants were to progress to kidney failure (see Fig. 2). We found that participants with advanced CKD at the time of first biopsy and high-risk *APOL1* alleles were more likely to progress to the composite eGFR decline outcome. Ethnicity, sex, race, presence of coexisting conditions, glomerular disease diagnosis, or medication adherence did not have statistically significant associations with progression to composite eGFR decline in this model. Participants with graduate degrees were less likely than participants with high school degrees/GEDs to progress to composite eGFR decline. However, we found no significant difference between participants with a high school degree/GED, less than a high school education, or a college degree in likelihood of progressing to composite eGFR decline. Stage 2 hypertension status at enrollment was found to correlate with progressing to composite eGFR decline while elevated blood pressure and stage 1 hypertension at enrollment were not found to correlate (see Table 3).

**Table 3:** Composite eGFR decline of children and young adults with glomerular disease Cox proportional hazards model output

Composite eGFR decline model	Hazard ratio (CI)
Age at glomerular disease onset (reference: 13–19)	
5–12	0.65 (0.39, 1.10)
20–29	1.25 (0.79, 1.97)
30–39	0.98 (0.61, 1.56)
Education (reference: high school)	
Less than high school	1.20 (0.65, 2.19)
2- or 4-year degree	1.09 (0.76, 1.57)
Graduate degree	0.44 (0.24, 0.84)
eGFR at biopsy (reference: eGFR 90 +)	
60–90	1.67 (1.06, 2.62)
30–60	3.72 (2.41, 5.74)
15–30	5.13 (2.87, 9.14)
< 15	4.45 (2.49, 11.91)
APOL1 risk (reference: low risk)	
High risk	1.90 (1.15, 3.15)
Unknown risk	1.11 (0.74, 1.67)
Hypertension status at enrollment (reference: normal blood pressure)	
Elevated blood pressure	0.82 (0.44, 1.52)
Stage 1 hypertension	1.52 (0.99, 2.34)
Stage 2 hypertension	3.26 (2.09, 5.06)

We defined composite eGFR decline as requiring chronic kidney replacement therapy or an eGFR < 15 mLs/min/1.73 m^2^ and/or extrapolated 40% reduction in eGFR since biopsy resulting in an observed eGFR < 60 mLs/min/1.73 m^2^. Refer to derived variables section of paper for education definition

## Discussion

Our data suggest that participants with adolescent-onset glomerular disease have similar rates of achieving sustained disease remission off treatment and similar rates of progression to kidney failure as participants with childhood, young adult, or adult-onset glomerular disease. While there was a trend towards higher rates of sustained disease remission and a trend towards lower rates of progression to composite eGFR decline seen in the unadjusted curves (Figs. 1 and 2) with younger ages of onset, the trend in unadjusted curves may be due to increased rates of hypertension seen in the cohorts with older ages of onset. As seen in Table 1, 5% of participants with onset of glomerular disease in childhood had stage 2 hypertension at the time of enrollment in CureGN compared to 24% with onset of glomerular disease in adulthood.

We further found that sex, race, and Hispanic ethnicity did not statistically significantly impact the likelihood of achieving sustained proteinuria remission off treatment or impact the likelihood of progression to composite eGFR decline. We did, however, find that participants with high-risk *APOL1* alleles were significantly less likely to experience sustained proteinuria remission off treatment and significantly more likely to experience progression to composite eGFR decline than people with low-risk *APOL1* alleles. Previous studies have suggested that individuals identifying as African–American/Black are 2–4 times more likely to progress to kidney failure compared to individuals identifying as White; however, these studies did not take into account genetic variants such as high-risk *APOL1* alleles and did not strictly pertain to glomerular disease patients [27]. Ongoing efforts to disentangle the impact of social constructs like race from biological factors like *APOL1* on progression of kidney failure remain important.

It also will be important to understand how prior therapeutic advances have impacted the CureGN cohort. For example, rituximab was used in 0.8% of our CureGN cohort from 2007 to 2011, 6.2% from 2012 to 2016, and 14.4% from 2017 to 2021. In all, roughly one-third of individuals with MCD and MN had exposure to rituximab, compared to just over eleven percent of individuals with FSGS. Describing trends in rituximab use across the various subgroups is a key focus for future research.

When evaluating the impact of the highest level of education attained on composite eGFR decline, we found that participants with a graduate degree were significantly less likely to progress to composite eGFR decline than participants obtaining a high school degree/GED. No significant difference was found when comparing other educational groups to the high school diploma/GED group. Using education as a proxy for socioeconomic status (SES), this data suggests that indeed participants with the highest SES have less risk of composite eGFR decline in this cohort. Higher levels of education have been associated with greater health literacy [28]. Given that both the health literature intended for patients as well as the information regarding clinical trial enrollment are often written at a level well above national literacy levels, people with higher levels of education may be best able to understand their disease and enroll in clinical trials if they are having insufficient response to conventional treatment [29].

Among participants across all age groups ~ 25% met the composite eGFR decline milestone within 5 years. Recently, multiple prescription drugs have received accelerated or full FDA approval for adults with kidney disease to delay progression, with a few such as sparsentan, a dual endothelin and angiotensin II receptor antagonist, iptacopan an alternative complement factor B inhibitor, and an oral targeted release formulation of budesonide recently coming to market for IgAN [30–33]. These drugs do not currently have approval in pediatric populations. Even therapies for supportive management of glomerular diseases such as the sodium-glucose cotransporter-2 inhibitors have been robustly tested in adults, but not in children [34]. Moreover, in some glomerular disease drug trials where individuals over age 65, but not adolescents, are eligible to participate, it is important to consider adolescents may better match the median age of disease diagnosis compared to older adults [11, 15, 35]. While our analysis does not address the use of specific drugs, our findings highlight the need for clinical trials in pediatric patients and effective drugs for all individuals with these four glomerular disease diagnoses.

Limitations of our study include the possibility that differences in clinical outcomes by age of glomerular disease onset are present, but that our study was underpowered to show these differences. While each of the cohorts had a similar median length of CureGN follow-up time, more than 25% of the cohort had less than 2 years of CureGN follow-up. Follow-up was supplemented by retrospective data collection in CureGN surveys to describe the period between diagnosis and enrollment, but longer post-enrollment follow-up duration may have revealed progression to composite eGFR decline or achievement of sustained proteinuria remission off treatment. Assessment of SES was limited to educational attainment as discussed above, with our assessment of educational attainment based on the education attained by study admission, which for young adults may not capture ultimate educational attainment, due to the patient enrolling prior to the age one would reasonably initiate a graduate degree. However, given the limited number of patients we believe this would affect, we do not believe this should significantly alter our SES assessment. The CureGN questionnaire does not ask about household income, and > 1/5 of the participants do not have a recorded zip code, thus education was the only measure used to investigate the relationship between SES and progression to kidney failure. This deficit is now being addressed in CureGN.

## Conclusions

Ultimately, our data did not show a statistically significant difference in clinical outcomes between adolescent onset glomerular disease and onset of glomerular disease at slightly younger and older ages. We saw ~ 25% of participants across all age groups reach the composite eGFR decline outcomes within 5 years. As more drug trials become available for adults with glomerular disease, it is important that we consider including people with both childhood and adolescent onset glomerular disease in these trials so they too benefit from new mechanisms to induce or sustain remission and limit progression.

## Supplementary Information

Below is the link to the electronic supplementary material.

Graphical abstract (PPTX 79 KB)

Supplementary file1 (DOCX 2311 KB)

## Data Availability

The data in this study were obtained from the Cure Glomerulonephropathy (CureGN) network where data sharing requires ancillary study approval and a data use agreement. The dataset may be requested from the CureGN Data Coordinating Center at The University of Michigan.
