# Supplementary material for: Comparing adolescent glomerular disease clinical outcomes to the clinical outcomes in childhood, young adult, and adult-onset glomerular disease in the CureGN database
Source: Pediatr Nephrol. 2024 Dec 27;40(6):1949–58. doi: 10.1007/s00467-024-06566-4 (PMC12031915; doi:10.1007/s00467-024-06566-4)
Supplement: Supplementary file 1 — Graphical abstract (PPTX 79 KB) [file 467_2024_6566_MOESM1_ESM.pptx]

## Slide 1
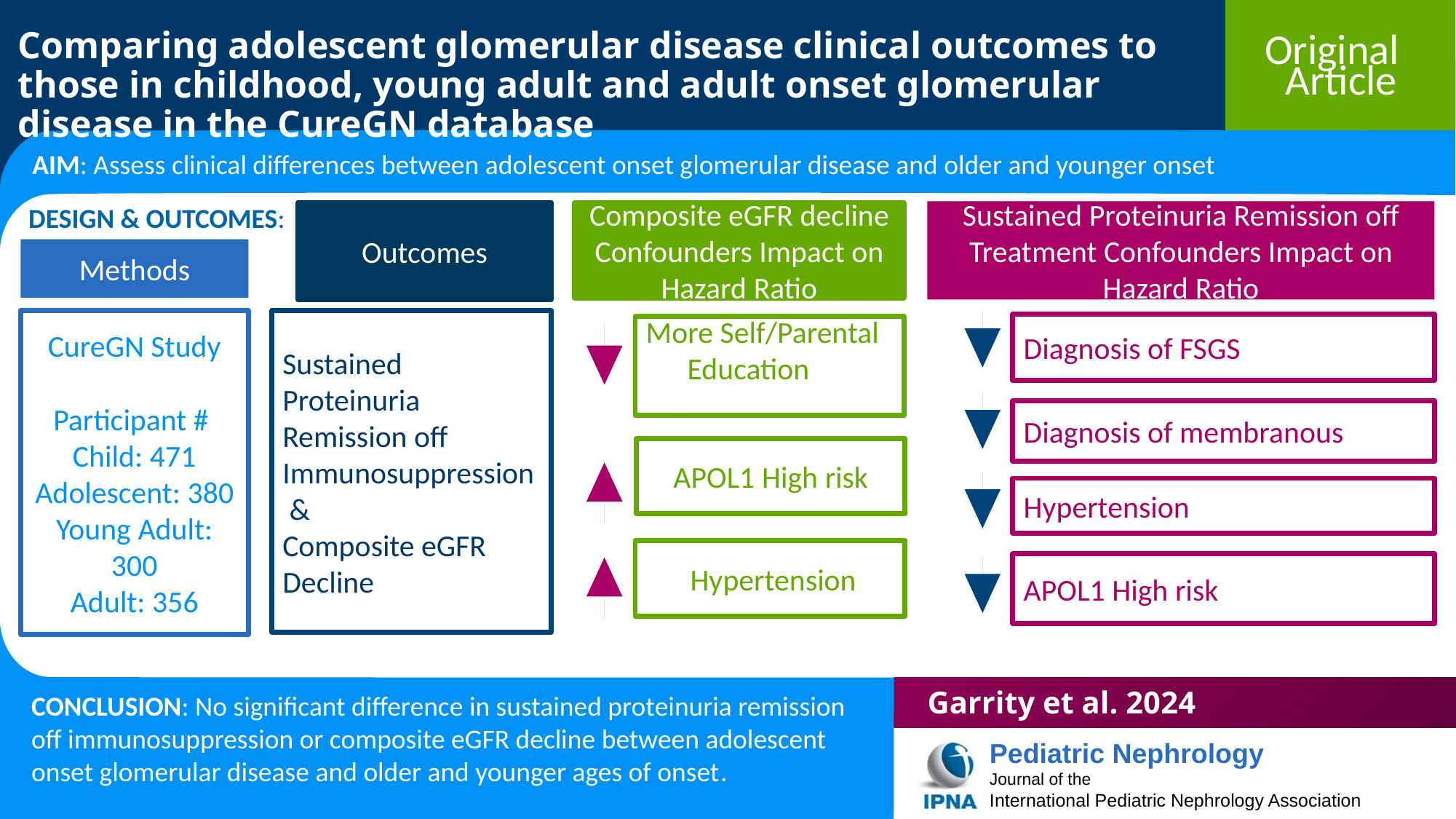

Comparing adolescent glomerular disease clinical outcomes to those in childhood, young adult and adult onset glomerular disease in the CureGN database
AIM: Assess clinical differences between adolescent onset glomerular disease and older and younger onset
DESIGN & OUTCOMES:
Sustained Proteinuria Remission off Treatment Confounders Impact on Hazard Ratio
Outcomes
Composite eGFR decline Confounders Impact on Hazard Ratio
Methods
CureGN Study
Participant #
Child: 471
Adolescent: 380
Young Adult: 300
Adult: 356
Sustained Proteinuria Remission off Immunosuppression &
Composite eGFR Decline
Diagnosis of FSGS
More Self/Parental EducationHTN HR:
Diagnosis of membranous
APOL1 High risk
Hypertension
 Hypertension
APOL1 High riskR: 0.c
Garrity et al. 2024
CONCLUSION: No significant difference in sustained proteinuria remission off immunosuppression or composite eGFR decline between adolescent onset glomerular disease and older and younger ages of onset.
