# Supplementary material for: Comparing adolescent glomerular disease clinical outcomes to the clinical outcomes in childhood, young adult, and adult-onset glomerular disease in the CureGN database
Source: Pediatr Nephrol. 2024 Dec 27;40(6):1949–58. doi: 10.1007/s00467-024-06566-4 (PMC12031915; doi:10.1007/s00467-024-06566-4)
Supplement: Supplementary file 2 — Supplementary file1 (DOCX 2311 KB) [file 467_2024_6566_MOESM2_ESM.docx]

S1


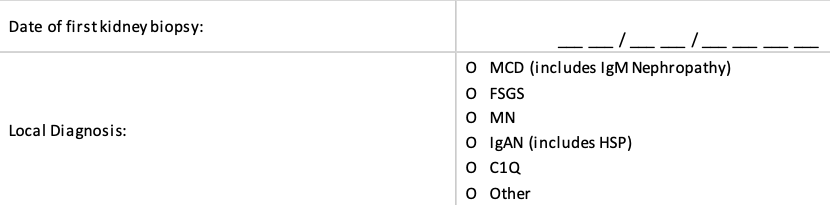

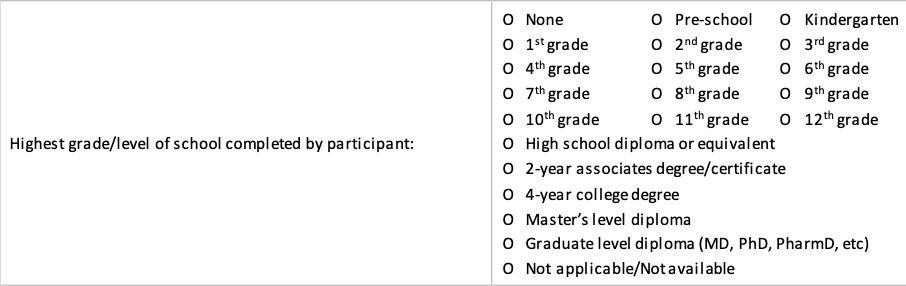

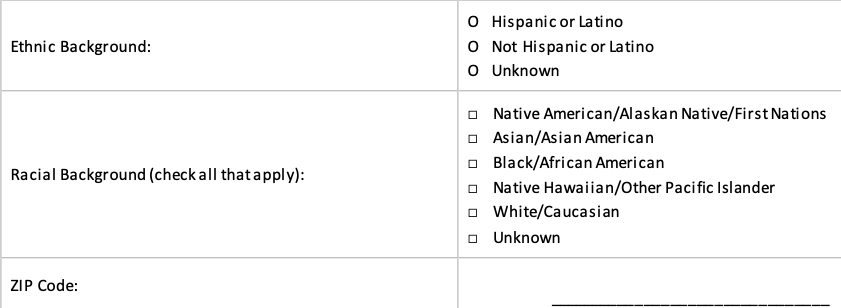


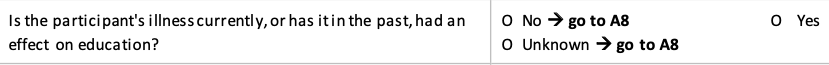


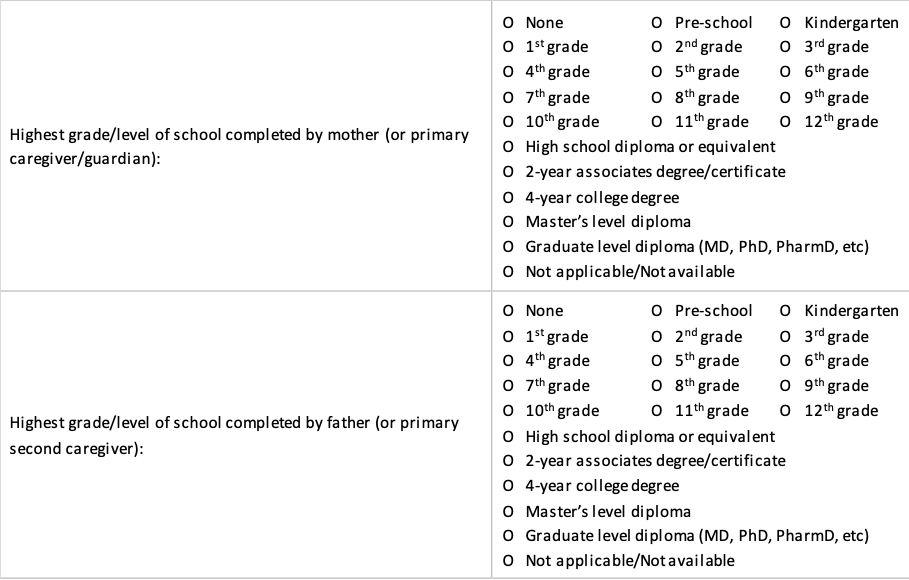


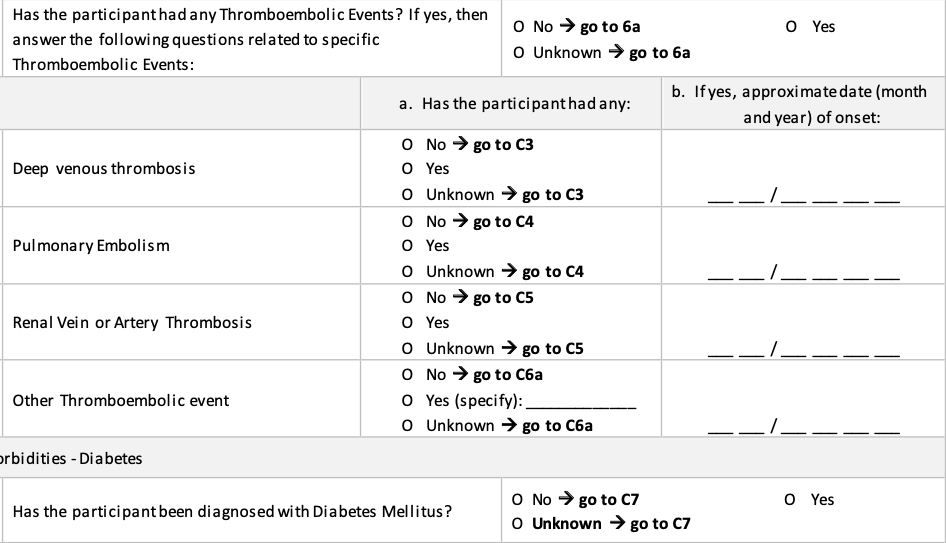


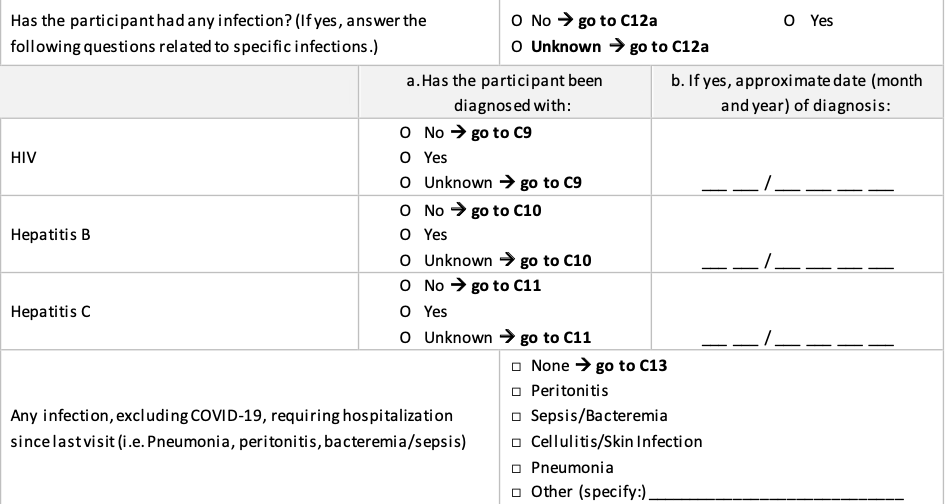


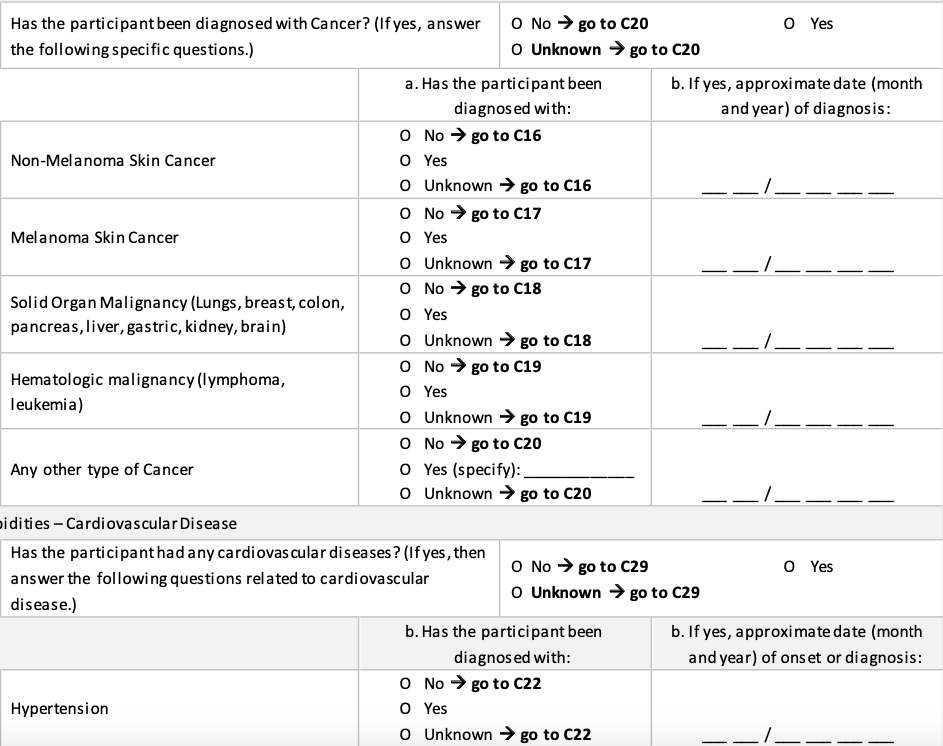


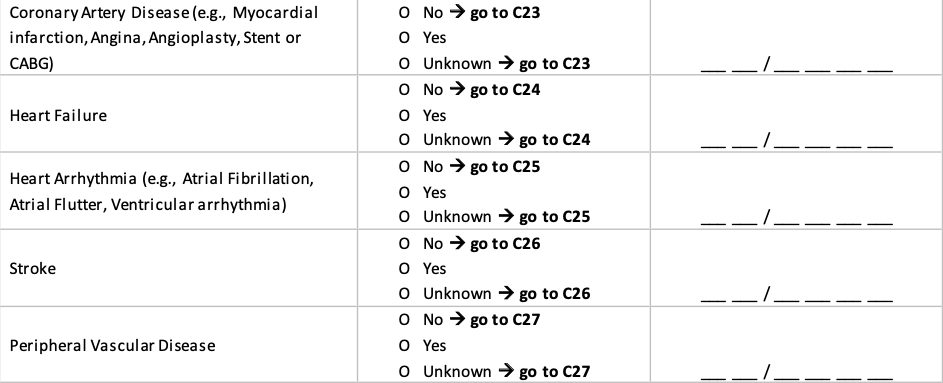


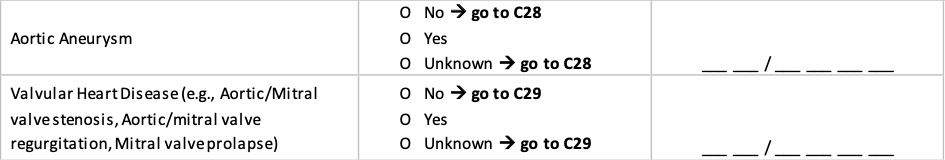


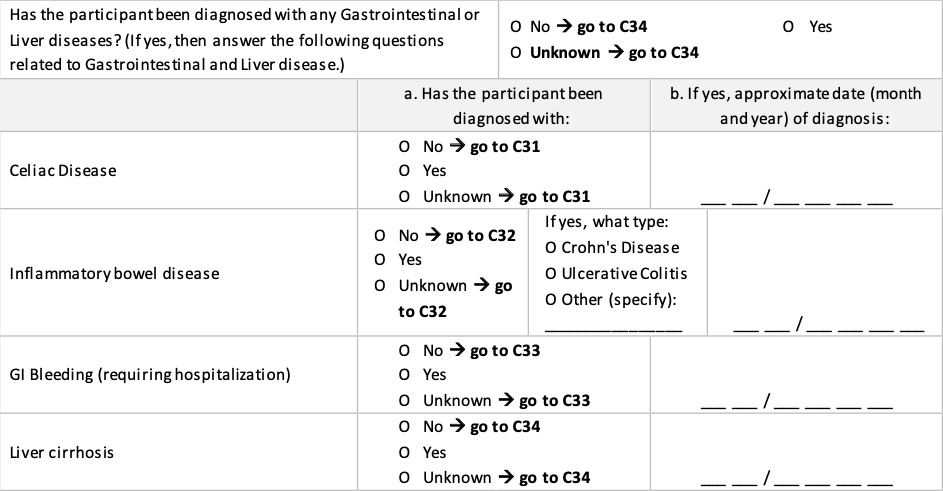


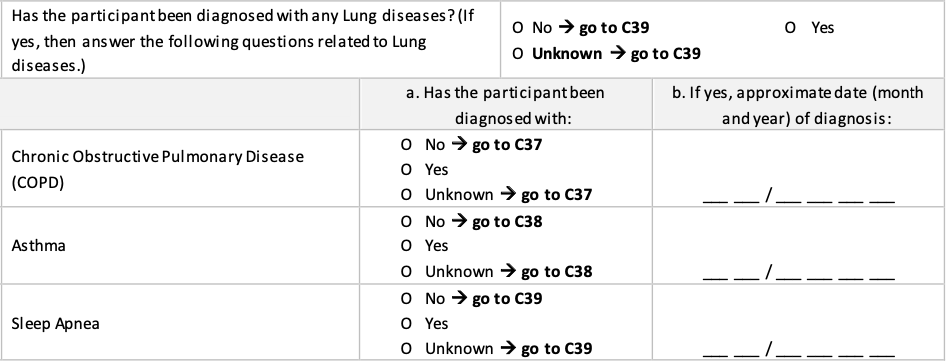


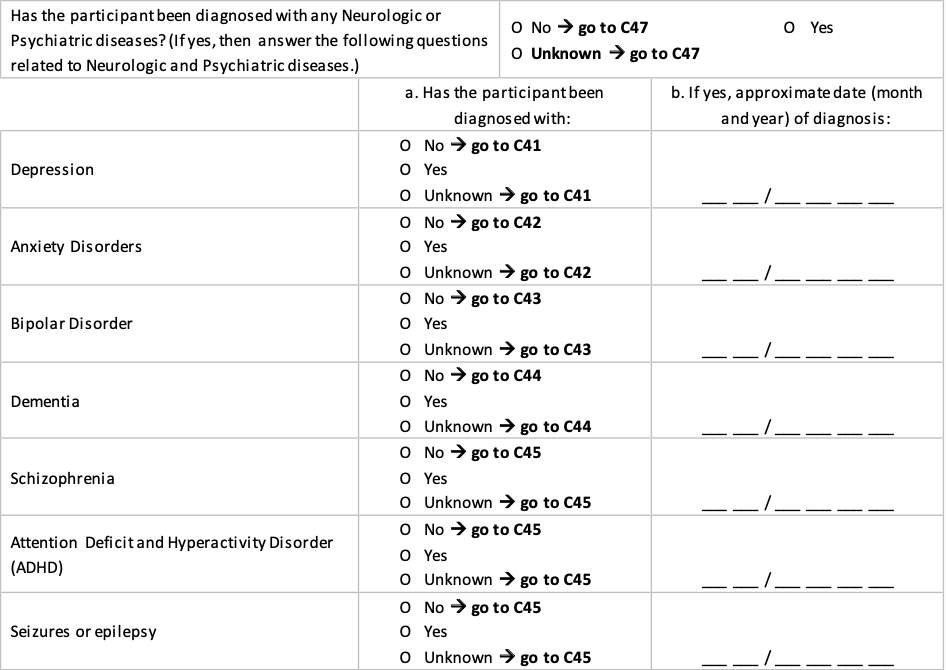


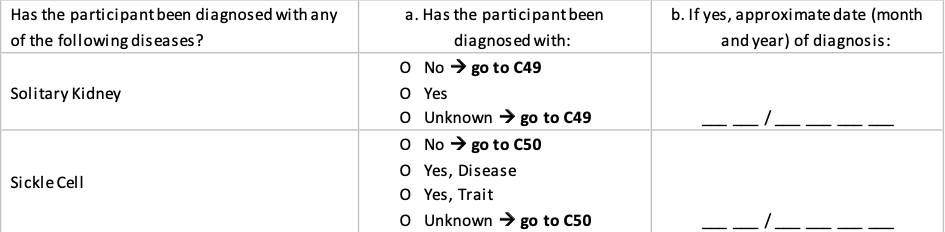


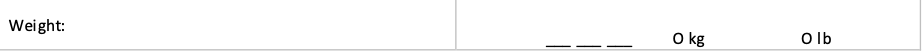


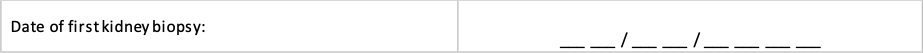


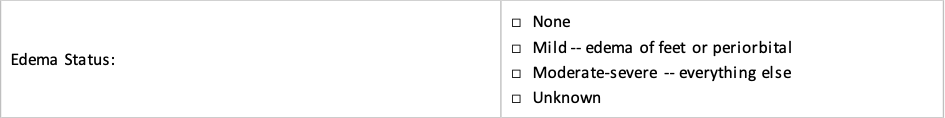


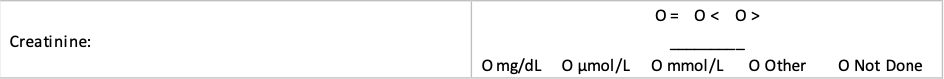


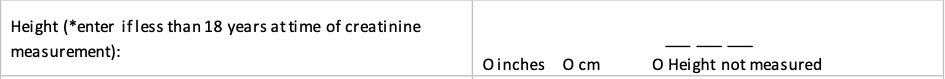


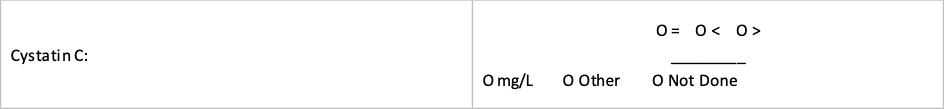


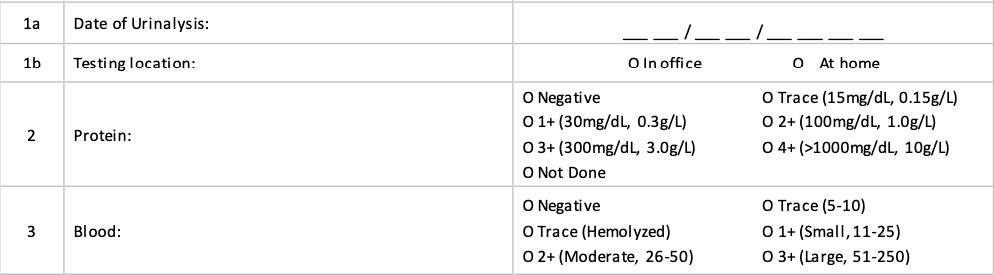


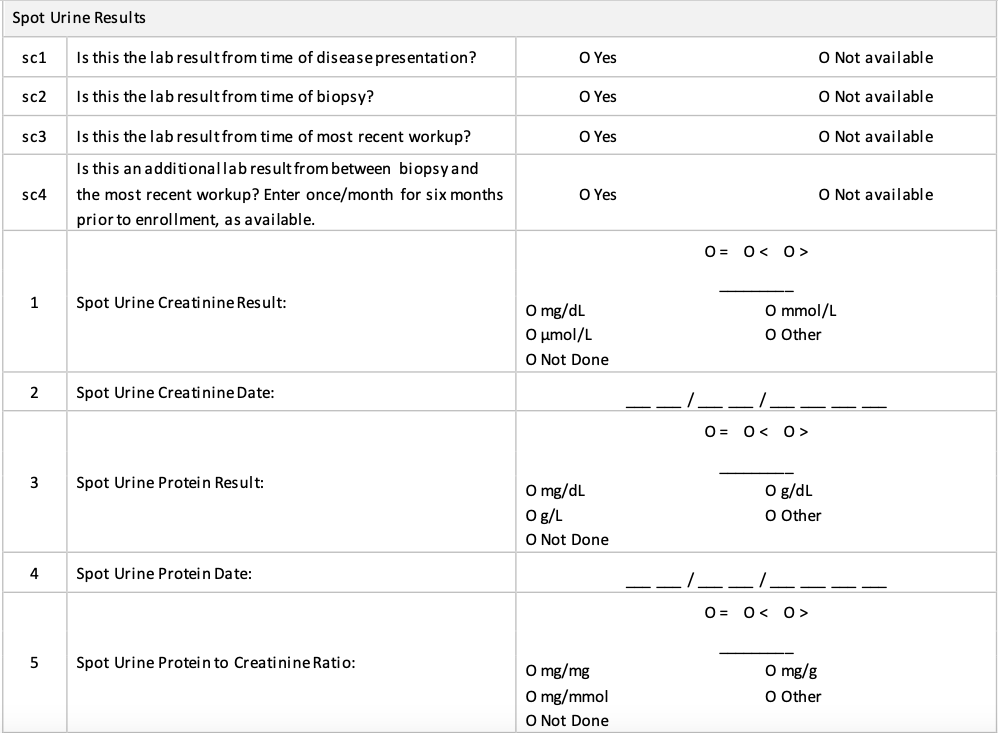


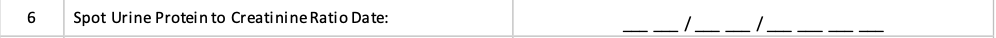


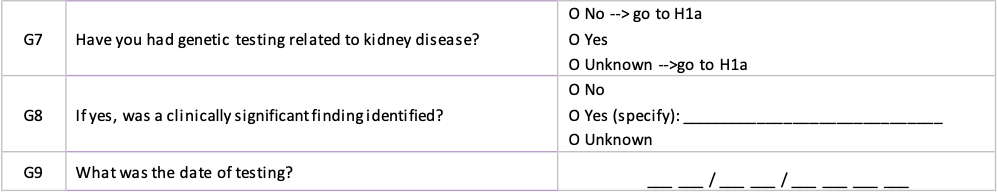


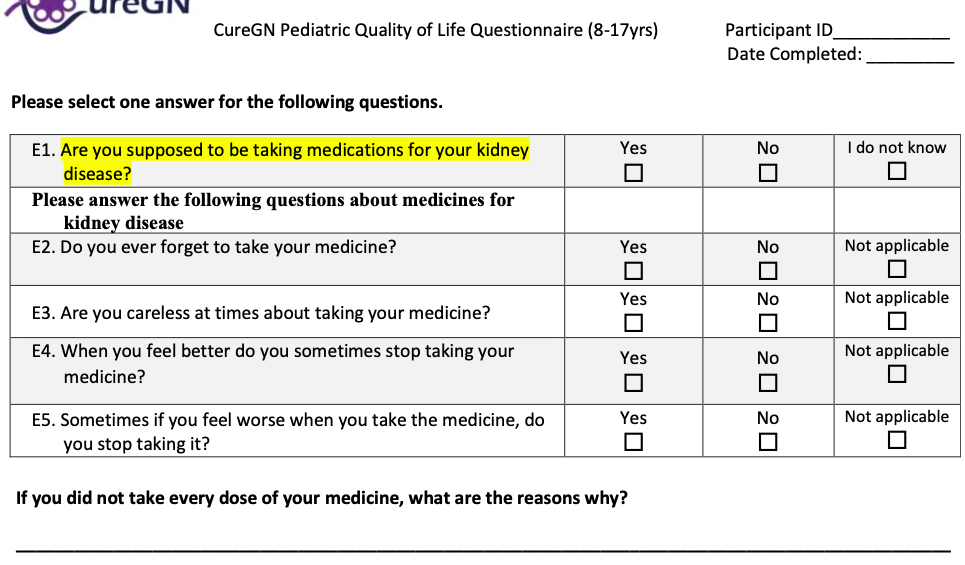


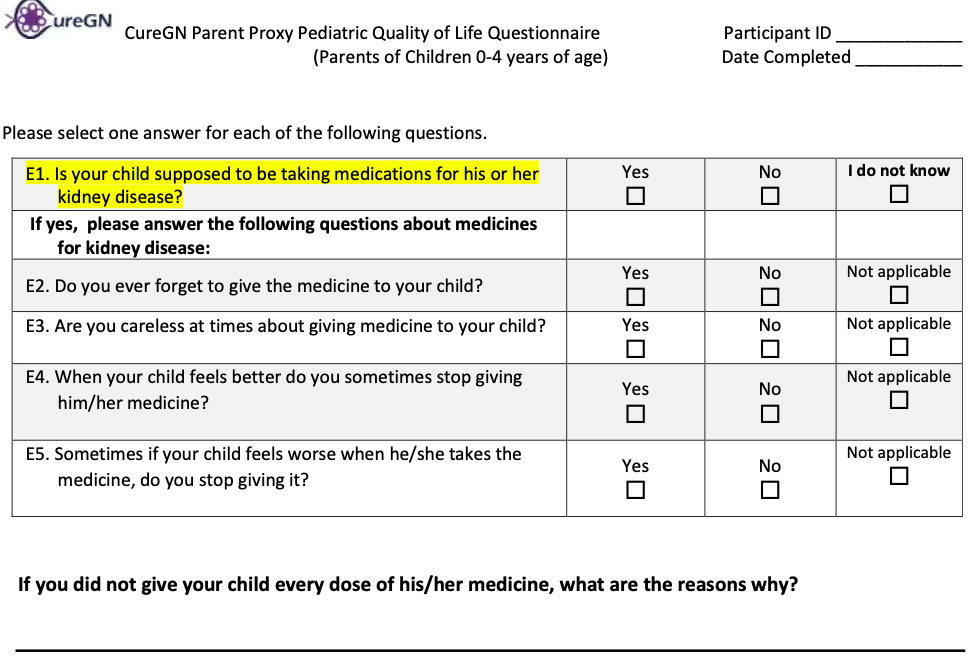


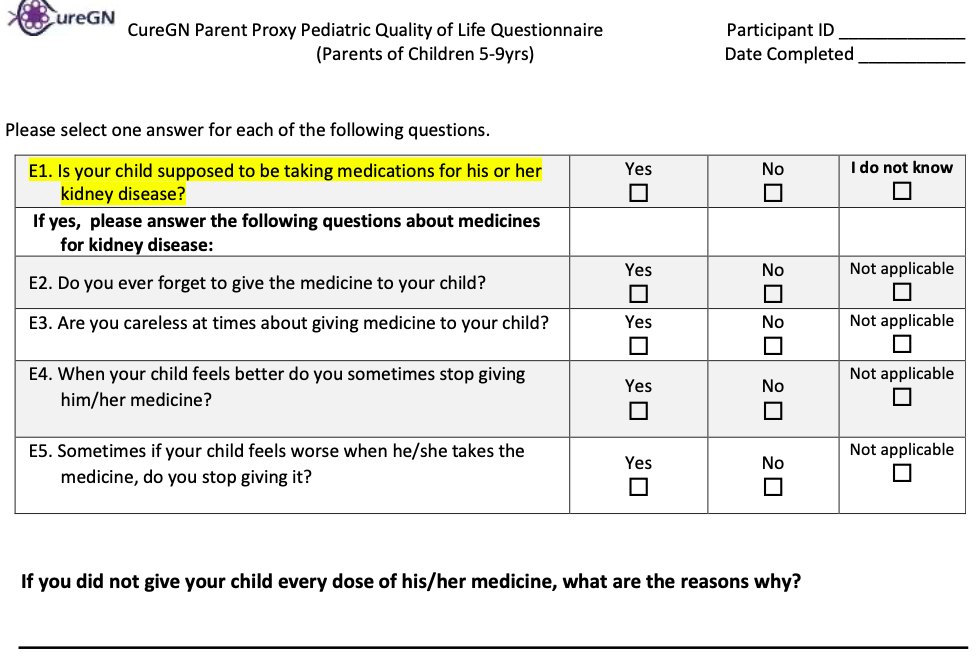

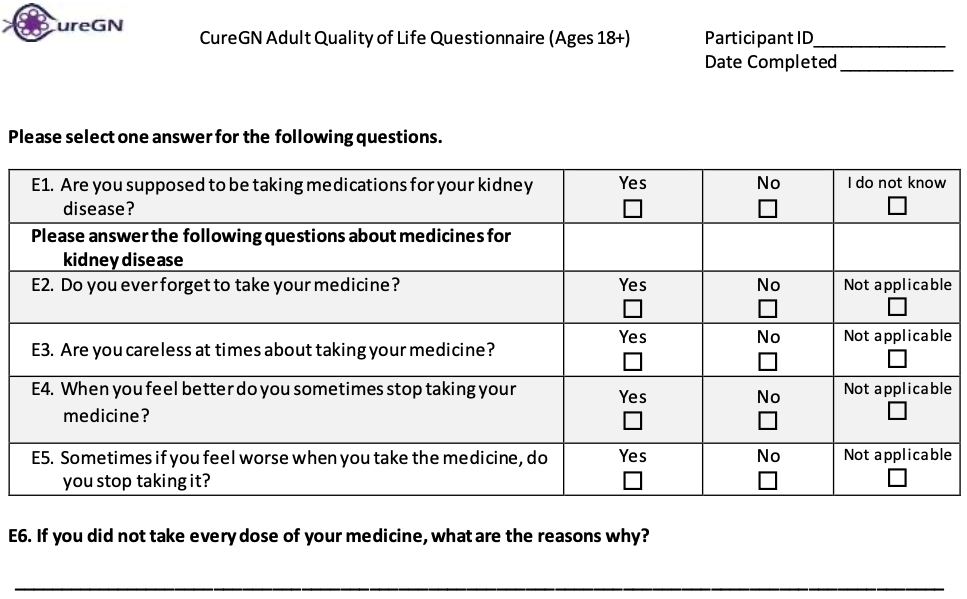


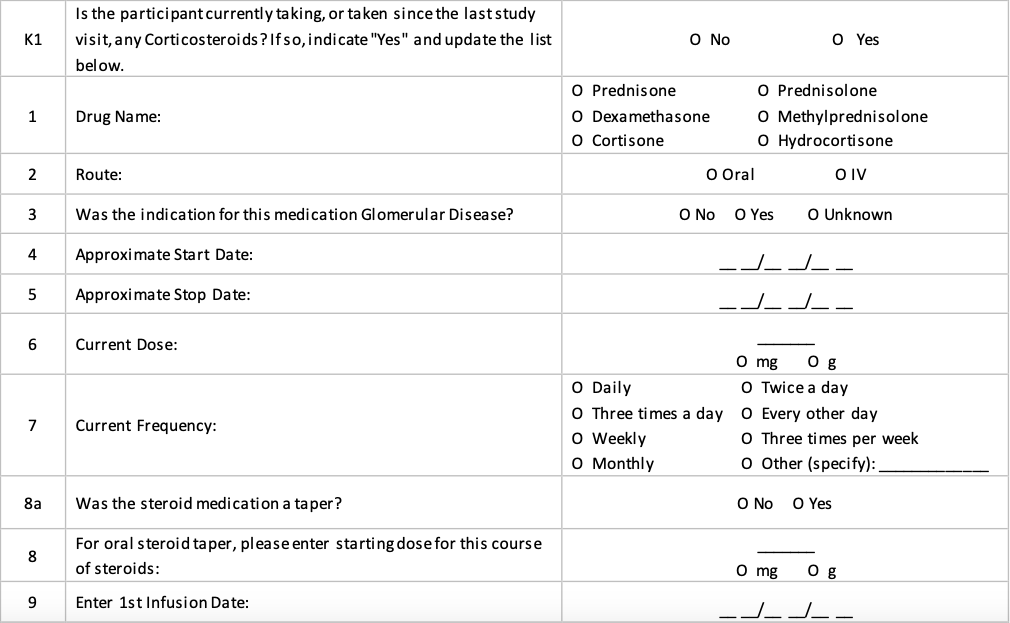


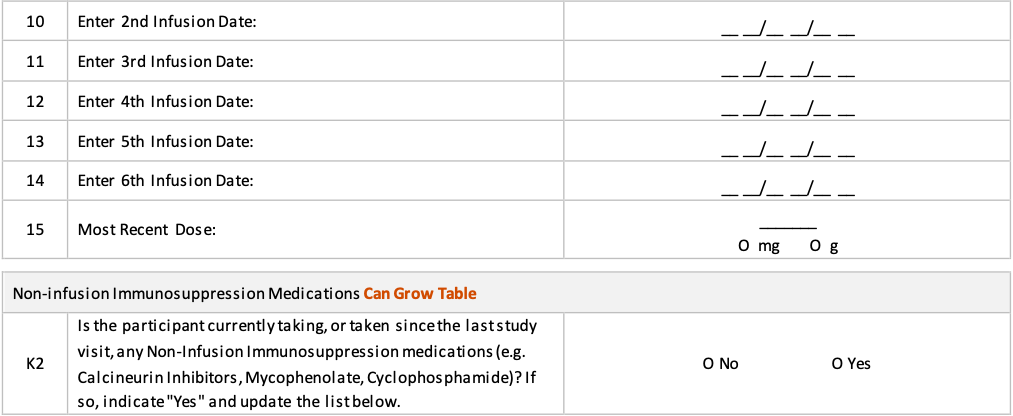


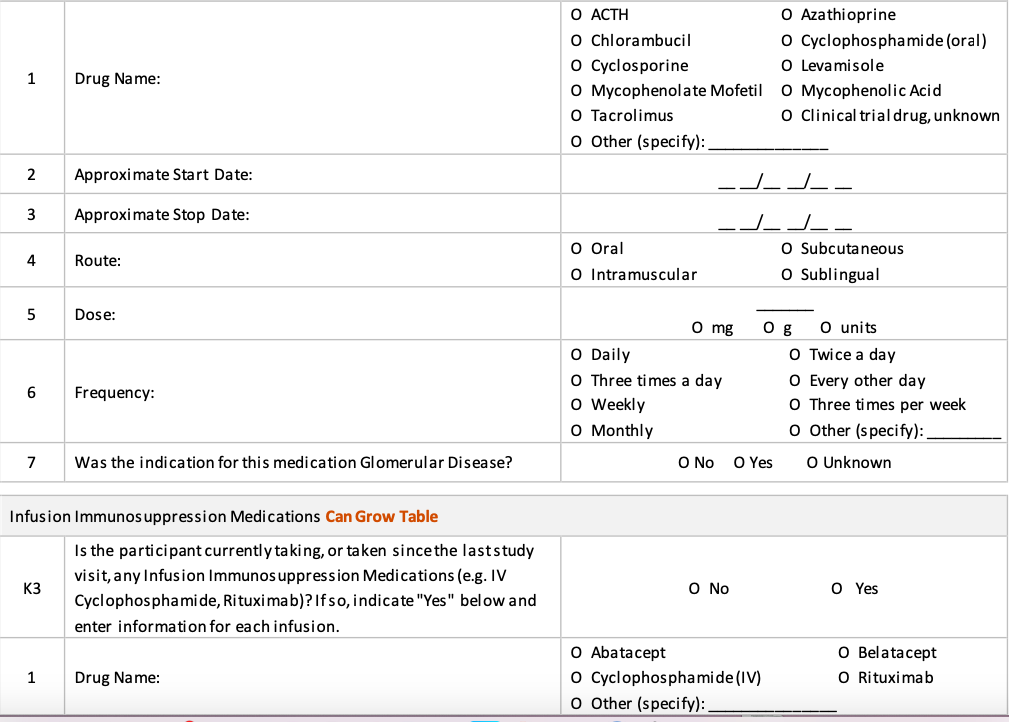


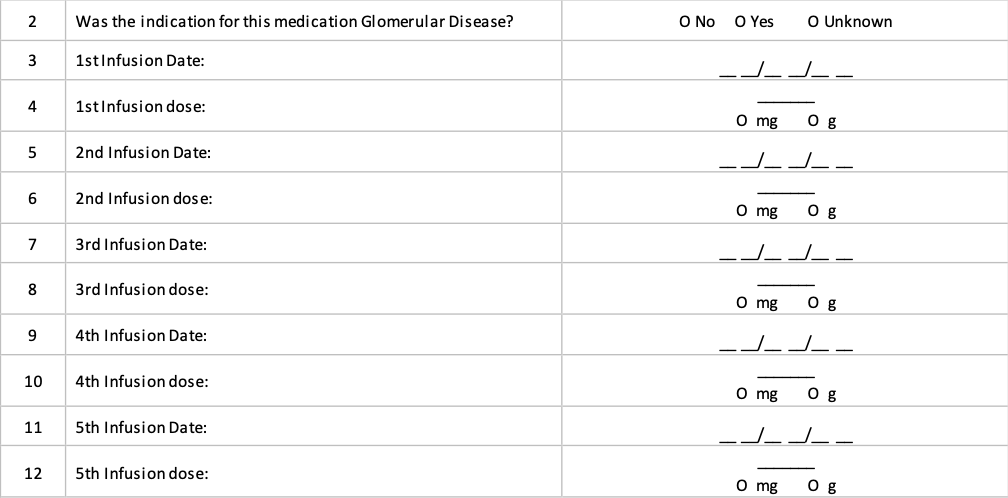


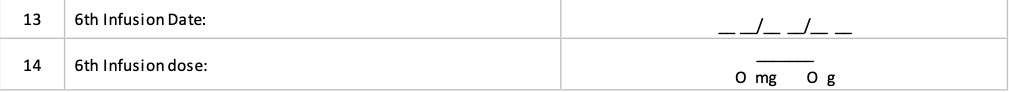


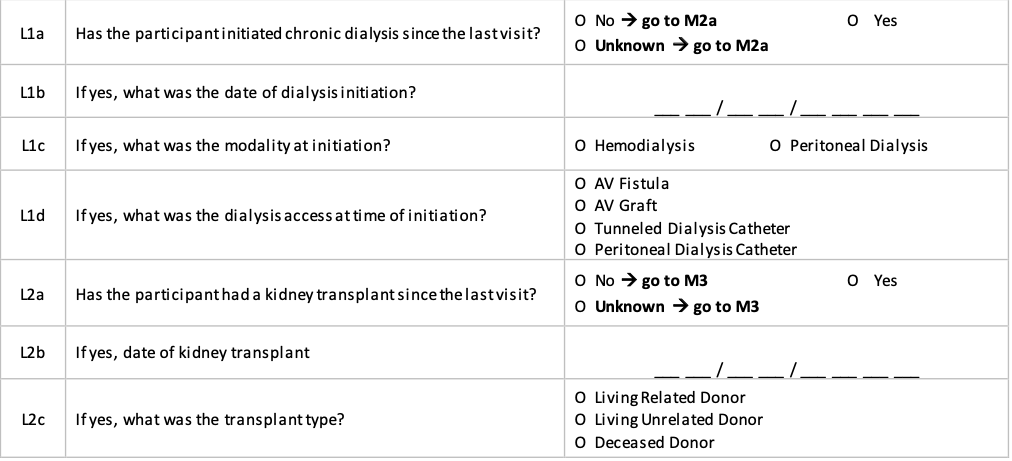


CureGN 1.0 asked about medication adherence using three questions, all of which were answered on a Likert scale from 1 to 5, with 1 meaning strongly disagree and 5 meaning strongly agree: “I took all doses of my disease medication”; “I missed or skipped at least one dose of my kidney disease medication”; “I was not able to take all of my kidney disease medication”. An answer of neutral to any disagreement (1, 2, or 3) to the first question or an answer to neutral or any agreement (3, 4, or 5) to the second or third questions was defined as medication nonadherence. After the CURE 2.0 revisions to the survey protocols, participants were asked two questions on medication adherence: “Are you careless at times about taking your medicine” and “Do you ever forget your medicine”. An affirmative response to either of these questions was defined as medication nonadherence.
